# Protective Effects of Punicalagin on Osteoporosis by Inhibiting Osteoclastogenesis and Inflammation *via* the NF-κB and MAPK Pathways

**DOI:** 10.3389/fphar.2020.00696

**Published:** 2020-05-15

**Authors:** Wei Wang, Jiaxiang Bai, Wenhao Zhang, Gaoran Ge, Qing Wang, Xiaolong Liang, Ning Li, Ye Gu, Meng Li, Wei Xu, Huilin Yang, Yaozeng Xu, Dechun Geng, Jun Zhou

**Affiliations:** ^1^Department of Orthopaedics, The First Affiliated Hospital of Soochow University, Suzhou, China; ^2^Department of Orthopedics, Soochow University Affiliated First People’s Hospital of Changshou City, Changshu, China; ^3^Division of Life Sciences and Medicine, The First Affiliated Hospital of USTC, University of Science and Technology of China, Hefei, China; ^4^Department of Orthopaedics, The Second Affiliated Hospital of Soochow University, Suzhou, China

**Keywords:** osteoporosis, bone loss, osteoclast, inflammation, punicalagin

## Abstract

Postmenopausal osteoporosis is a worldwide disease characterized by reduced bone mineral density and increased fracture risk. Inflammatory bone loss due to excessive osteoclast bone resorption is significant in the pathogenesis and development of osteoporosis. Punicalagin (PUN) is a pomegranate fruit derivative and has potential anti-inflammatory effects. However, the effect of PUN on osteoporotic bone loss has yet to be clarified. In this study, we investigated the effect of PUN on RANKL-induced osteoclast formation and bone resorption *in vitro*, as well as its potential therapeutic effect on ovariectomized-induced bone loss *in vivo*. PUN was demonstrated to suppress osteoclast formation and bone resorptive function dose-dependently, while osteoclast-specific genes were also downregulated by PUN. In vivo micro-CT and histopathological staining showed that the OVX procedure led to significant bone loss characterized by decreased bone parameters and increased osteoclast numbers, while PUN treatment dramatically prevented these changes. Furthermore, PUN treatment effectively inhibited proinflammatory cytokine expression *in vitro*. Mechanistically, PUN maintained bone mass *via* suppressing nuclear factor κB (NF-κB) and mitogen‐activated protein kinase (MAPK) signaling pathway activation. Collectively, our observations provide evidence that PUN is a potential candidate for the treatment of osteoporosis.

## Introduction

Postmenopausal osteoporosis is a systemic metabolic bone disease that affects more than 200 million women worldwide (Zhao K. et al., 2019). Postmenopausal osteoporosis caused by dysfunctions in bone remodeling causes low bone mineral density and microstructural changes, which decrease bone strength and increase bone fragility, leading to increased fracture risk (Chen et al., 2018; Zhao X. et al., 2019). It has been reported that one of three women older than 50 years will undergo osteoporotic fractures, which has a significant impact on the patients’ daily activities and quality of life, as well as significant economic costs for both individuals and society (Eriksen et al., 2014; Lin et al., 2016; An et al., 2019). Moreover, postmenopausal osteoporosis further increases as the population ages. Estrogens, estrogen receptor modulators, calcitonin, and bis-phosphonates are the current medications for bone loss. Anti-osteoporotic drugs are efficacious, but unfortunately, they increase the potential risk of breast cancer, myocardial ischemia, thromboembolic disease, and atypical femur facture (Eriksen et al., 2014; Black and Rosen, 2016; Compston et al., 2019; Zhao K. et al., 2019). Denosumab, a monoclonal antibody, can inhibit bone absorption by inhibiting the differentiation and activation of osteoclasts, but skin rash and infection occur more frequently, as well as the effect on bone turnover is rapidly reversible after drug discontinuation (Compston et al., 2019). Therefore, it is desirable to discover more efficacious and safer drugs for osteoporosis therapy.

At menopause, the sharp decrease in estrogen levels leads to enhanced bone resorption owing to increased osteoclastogenesis (Sun et al., 2010; Li et al., 2019). Osteoclasts originate from bone marrow monocytes/macrophages (Chen et al., 2019). Estrogen deficiency induces the activation of receptor activator of nuclear factor κB (NF-κB) ligand (RANKL), the key molecule required for osteoclast differentiation, which can bind with receptor activator of NF-κB (RANK) on preosteoclast membranes and stimulate the NF‐κB and mitogen‐activated protein kinase (MAPK) pathways for osteoclast differentiation and formation (Chen et al., 2018; Jiang et al., 2019; Shang and Wu, 2019). Then, nuclear factor of activated T cells cytoplasmic 1 (NFATc1), the main transcription factor that regulates osteoclast differentiation, will be triggered to stimulate preosteoclast maturation (Jiang et al., 2019; Zhao K. et al., 2019). Maturational osteoclasts, which are characterized by tartrate-resistant acid phosphatase (TRAP) expression, can generate actin-bound sealing zones for attaching to the surface of bone, and osteoclasts then release proteolytic enzymes, such as cathepsin K (CTSK), which promote resorption pit formation (Lee et al., 2019; Zhao X. et al., 2019). Excessive osteoclastogenesis and bone resorption result in changes in trabecular bone mass and microstructure, leading to the potential risk of fracture; this indicates the need to find therapeutic approaches to prevent osteoporosis (Eriksen et al., 2014; Chen et al., 2019; Jacome-Galarza et al., 2019). Osteoporosis is also considered a chronic inflammatory disease, as several increased proinflammatory cytokines, including tumor necrosis factor (TNF-α), interleukin (IL)-1β, and IL-6, may regulate RANKL expression (Zhao et al., 2016; Michalski and McCauley, 2017; Zeng et al., 2019). Therefore, inhibiting proinflammatory osteoclastogenesis activation could be a promising strategy for the discovery of antiosteoporosis drugs. Targeting osteoclasts with natural products has been confirmed to be an important therapeutic approach for osteoporosis (Newman and Cragg, 2016).

Punicalagin (2,3-(S)-hexahydroxydiphenoyl-4,6-(S)-gallagyl-d-glucose, PUN) is a polyphenol that is found mainly in the pomegranate fruit (Johanningsmeier and Harris, 2011). PUN has many important pharmacological effects, such as anti-inflammatory, antitumor, and antioxidative properties (Johanningsmeier and Harris, 2011; Atrahimovich et al., 2018; Seo et al., 2018). Previous evidence has confirmed that PUN treatment has beneficial effects on some diseases, including memory impairment (Kim et al., 2017), Alzheimer’s disease (Seo et al., 2018), and endometritis (Lyu et al., 2017), due to its suppression of MAPK and NF-κB activation, but the effect of PUN on osteoporotic bone loss has yet to be shown. Given the potential anti-inflammatory function and other potential therapeutic applications of PUN, we hypothesized that PUN may suppress osteoclastogenesis to attenuate osteoporotic bone loss.

In the current study, we demonstrated that PUN treatment inhibits RANKL-induced osteoclast formation *in vitro* and attenuates ovariectomized-induced bone destruction *in vivo*. We further demonstrated that PUN treatment effectively decreased proinflammatory cytokine expression levels. Mechanistically, we found that PUN suppressed the NF-κB and MAPK signaling pathways.

## Materials and Methods

### Drugs and Reagents

PUN (purity ≥98%) was obtained from Sigma-Aldrich (St. Louis, USA) and dissolved in phosphate-buffered saline (PBS). PBS was used as the vehicle control. Recombinant RANKL was purchased from R&D Systems (Minneapolis, USA). Minimal essential medium (DMEM/high glucose) and fetal bovine serum (FBS) were purchased from HyClone (Logan, USA). LPS (*E. coli* 026: B6) was obtained from Sigma-Aldrich. Primary antibodies to phospho-NF-κB p65 (#3033), phospho-IκBα (#2859), NF-κB p65 (#8242), IκBα (#4814), phospho-ERK (#4370), phospho-p38 (#4511), phospho-JNK (#4668), ERK (#4695), p38 (#8690), and JNK (#9252) were purchased from Cell Signaling Technology (Boston, USA). Anti-rabbit and anti-mouse HRP-conjugated secondary antibodies were obtained from Multi Sciences (Shanghai, China). A CCK-8 assay kit was purchased from ApexBio (Boston, USA).

### Cell Lines

The murine macrophage cell line RAW 264.7 was used in the current study as it is widely used in osteoclastic experiments (Liang et al., 2019; Zeng et al., 2019). RAW 264.7 cells were obtained from the Cell Bank of the Chinese Academy of Sciences (Shanghai, China). DMEM supplemented with 10% FBS and penicillin/streptomycin antibiotics was used to culture the cells. A humidified incubator at 5% CO_2_ and 37°C was used for cell culture.

### Cytotoxicity Assay

The cytotoxicity of PUN was tested using a CCK-8 kit. Briefly, RAW 264.7 cells (2 × 10^3^) were reseeded into 96-well plates and left overnight to adhere. After 12 h, the cells were treated with various concentrations of PUN for 48 h. A microplate reader (BioTek, Vermont, USA) was used to measure the optical density (OD) according to the spectrophotometric absorbance at 450 nm.

### In Vitro Osteoclast Formation Assay

RAW 264.7 macrophages were reseeded into 48-well plates (1 × 10^4^ cells per well). Cells were pretreated with various concentrations of PUN (0, 5, 10, 20, and 50 μM) for 6 h and then induced in medium containing 50 ng/ml RANKL and various concentrations of PUN for 5 days. The medium was changed after incubation for 2 days. Then, the cells were fixed with a 4% formaldehyde solution for 15 min and stained with a TRAP staining kit (Sigma-Aldrich) after washing with PBS. The osteoclasts (nuclei ≥ 3) in each well were counted at 320× magnification using an inverted microscope (Zeiss, Dresden, Germany).

### F-Actin Ring Structure Formation Observation

An F-actin ring formation assay was performed according to the method described (Hu et al., 2017). In brief, cells were reseeded in 48-well plates (1 × 10^4^ cells/well). After adherence, the cells were treated with different concentrations of PUN (0, 5, 10, 20, and 50 μM) and RANKL (50 ng/ml). The medium was changed every 2 days. After 4 days, the cells were fixed with a 4% formaldehyde solution for 15 min and permeabilized with 0.2% Triton-X for 10 min after three washes. After that, the cells were blocked with QuickBlock™ blocking buffer (Beyotime, Shanghai, China) for 45 min and further stained with phalloidin for 1 h at 37°C. Finally, the cells were stained with DAPI for 3 min, and the F-actin ring and nuclei were then photographed using a fluorescence microscope (Zeiss, Dresden, Germany) at 320× magnification.

### Pit Formation Assay

A pit formation assay was performed to determine the effect of PUN on osteoclast function as described previously (Wang et al., 2018). RAW 264.7 cells were reseeded in 24-well Corning Osteo Assay Plates (Corning, NY, USA) at a density of 3 × 10^4^ cells per well. Next, the cells were treated with PUN (0, 5, 10, 20, and 50 μM) and RANKL (50 ng/ml). After 4 days, a supersonic wave was used to remove the cells, and a bright microscope (Zeiss, Dresden, Germany) was used to capture images of the resorptive pits. The percent resorptive areas were quantified by Image J software (National Institutes of Health, Bethesda, USA).

### Quantitative Real-Time PCR

RAW 264.7 cells were reseeded in 6-well plates (5 × 10^5^ cells per well) and stimulated with 50 ng/ml RANKL in the presence of PUN (0, 10, and 50 μM) for 24 h. Total RNA was isolated by using TRIzol reagent (Beyotime, Shanghai, China), and 1 μg of total RNA template was then used for cDNA synthesis using dNTP mix and RNase-free H_2_O (Abcam, Cambridge, UK). SYBR Green PCR Master Mix (Yeasen, Shanghai, China) was used for relative quantitative real-time PCR (qPCR) amplification. Glyceraldehyde 3-phosphate dehydrogenase (GAPDH) was used as an internal control. The mouse primer gene sequences are shown in **Table 1**.

**Table 1:** Sequences of mouse primers used in qRT-PCR.

Primers for qPCR		
Acp5	F	TGTGGCCATCTTTATGCT
	R	GTCATTTCTTTGGGGCTT
MMP9	F	CGTGTCTG GAGATTCGACTTGA
	R	TTGGAAACTCACACGCCAGA
CTSK	F	CTTCCAATACGTGCAGCAGA
	R	TCTTCAGGGCTTTCTCGTTC
OSCAR	F	GGAATGGTCCTCATCTGCTT
	R	GGAATGGTCCTCATCTGCTT
ATP6v0d2	F	GACCCTGTGGCACTTTTTGT
	R	GTGTTTGAGCTTGGGGAGAA
DC-STAMP	F	AAAACCCTTGGGCTGTTCTT
	R	AATCATGGACGACTCCTTGG
IL-1β	F	ACTCATTGTGGCTGTGGAGA
	R	TTGTTCATCTCGGAGCCTGT
IL-6	F	TCGTGGAAATGAGAAAAGAGTG
	R	AGTGCATCATCGTTGTTCATACA
TNF-α	F	CTGAGGTCAATCTGCCCAAGTAC
	R	CTTCACAGAGCAATGACTCCAAAG
iNOS	F	ATCTTTGCCACCAAGATGGCCTGG
	R	TTCCTGTGCTGTGCTACAGTTCCG

### Western Blot Assay

RAW 264.7 cells were reseeded in 6-well plates (5 × 10^5^ cells per well) and stimulated with 50 ng/ml RANKL and different concentrations of PUN. Then, radioimmunoprecipitation (RIPA) lysis buffer was used to lyse cells to harvest protein. Equal amounts of proteins were separated using SDS-polyacrylamide gel electrophoresis and then transferred to nitrocellulose membranes. The membranes were blocked with QuickBlock™ blocking buffer (Beyotime) before incubation with primary antibodies at 4°C. After overnight shaking, the membranes were washed with TBS-Tween and then incubated with horseradish peroxidase (HRP)-conjugated secondary antibodies. Enhanced chemiluminescence (ECL; Sigma-Aldrich) was used to detect the antibodies, and the relative gray level was analyzed using Image Lab software version 3.0 (Bio-Rad).

### Ovariectomy-Induced Osteoporosis Mouse Model

All animal experimental procedures were performed with the approval of the Ethics Committee of the First Affiliated Hospital of Soochow University. Forty ten-week-old female C57BL/6 mice were separated randomly into four groups (n = 10/group): sham operation group (ovaries were only exteriorized but not resected + PBS treatment), OVX group (bilateral ovariectomy was carried out to induce osteoporosis + PBS treatment), low PUN treatment group (bilateral ovariectomy was carried out to induce osteoporosis + 20 mg/kg PUN treatment), and high PUN treatment group (bilateral ovariectomy was carried out to induce osteoporosis + 50 mg/kg PUN treatment). All animals were under nembutal anesthesia during the operations, and all mice were allowed to recover for one week. Then, the mice in the PUN-treated group were administered by gavage with PUN five times per week for 4 weeks. The mice in the sham and OVX groups were treated with PBS as a control.

### Micro-CT Analysis

After fixation in 10% neutral buffered formalin for 24 h, mouse femurs were analyzed by a Skyscan 1176 micro-CT (Aartselaar, Belgium). The femurs (n = 5) were scanned using the following settings: 9 μm equidistant resolution, 50 kV and 200 μA energy X-ray. The images were then reconstructed to perform three‐dimensional (3D) histomorphometric analysis with NRecon software (Skyscan micro-CT, Aartselaar, Belgium). SkyScan software was used to analyze bone mineral density (BMD, mg/cm^3^), bone volume (BV, mm^3^), bone volume per tissue volume (BV/TV, %), connectivity density (Conn.Dn, 1/mm^3^), trabecular number (Tb.N, 1/mm), trabecular separation (Tb.Sp, mm), and trabecular thickness (Tb.Th, mm).

### Bone Histomorphometry Analysis

Femurs were decalcified in 10% ethylenediaminetetraacetic acid at 37°C for 4 weeks and then embedded in paraffin. Next, histological slices were prepared (Leica 2135, Germany), and hematoxylin and eosin (H&E) staining and TRAP activity staining were performed. Then, the tissue sections were dewaxed with xylene and subjected to gradient hydration. Section images were acquired using an Axiovert 40C optical microscope (Zeiss, Germany).

### Statistical Analysis

The data are shown as the mean ± standard deviation (SD). All experiments were performed at least three times independently. One-way analysis of variance (ANOVA) was used to measure the intergroup variation, and Bonferroni’s multiple comparison test was used to compare between groups by GraphPad Prism version 8.0. A *p* value <0.05 was considered statistically significant.

## Results

### PUN Suppressed Osteoclast Differentiation

**Figure 1:**
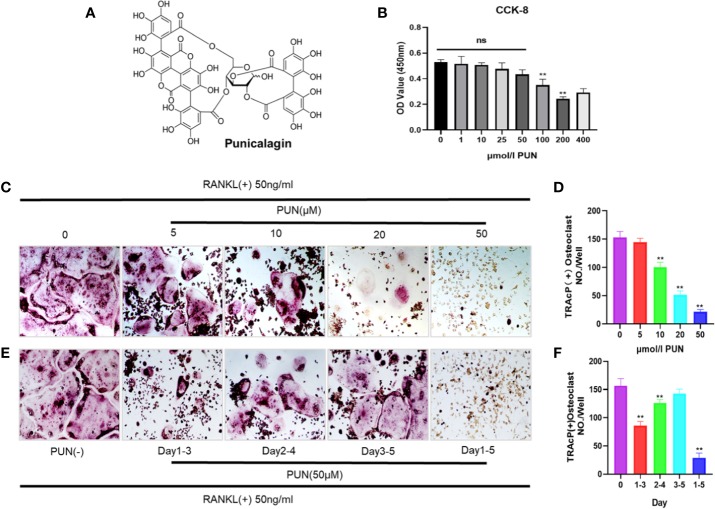
PUN suppressed RANKL-induced osteoclastogenesis. **(A)** The chemical structure and formula of PUN. **(B)** CCK-8 analysis was performed with various concentrations of PUN in RAW 264.7 macrophages for 48 h. PUN treatment at concentration 50 μM had no effect on cell viability. **(C)** After pre-treatment with various concentration of PUN (0, 5, 10, 20, and 50 μM) for 6 h. RAW 264.7 macrophages were stimulated with 50 ng/ml RANKL for 5 days. TRAP staining was used to determine osteoclast formation and differentiation, which showed that PUN inhibited osteoclastogenesis dose-dependently. **(D)** Quantification of TRAP-positive multinucleated cells (nuclei≥3). **(E)** After pre-treatment with 50 μM PUN for 6 h, RAW 264.7 macrophages were stimulated with 50 ng/ml RANKL and 50 μM PUN for the indicated days during osteoclastogenesis. **(F)** Quantification of TRAP-positive multinucleated cells showing that PUN inhibited osteoclastogenesis in different time periods. All bar graphs are presented as mean ± SD. **p < 0.01 compared with control group, n = 3 per group. NS, not statistically significant.

**Figure 1A** shows the chemical structure and formula of PUN. First, we performed a CCK-8 analysis to determine the cytotoxicity of PUN, and the results showed that PUN (5–50 µM) did not affect cell viability (**Figure 1B**). Then, to determine the effect of PUN on osteoclast formation, RAW 264.7 macrophages were reseeded in 48-well plates and treated with 50 ng/ml RANKL in the presence or absence of various concentrations of PUN as indicated. Apparent osteoclast differentiation and increased TRAP multinucleated osteoclasts were observed after RANKL induction (**Figure 1C**). However, treatment with PUN reduced RANKL-induced osteoclastogenesis in a dose-dependent manner (**Figures 1C, D**). Then, cells were treated with 50-μM PUN at different time phases to identify the affected osteoclast differentiation stage (**Figure 1E**). PUN obviously exerted its suppressive effect in the early stage (days 1–3) of osteoclastogenesis (**Figure 1F**). Osteoclastogenesis inhibition by PUN was not due to cytotoxicity, as PUN (5–50 µM) did not affect cell viability. These results indicated that PUN possesses the ability to suppress osteoclast differentiation in the RANKL induction system without causing cytotoxic effects.

### PUN *A*ffected F-*A*ctin *R*ing *F*ormation and *I*nhibited *B*one-*R*esorbing *A*ctivity and *O*steoclast-*S*pecific *Gene Expression*

**Figure 2:**
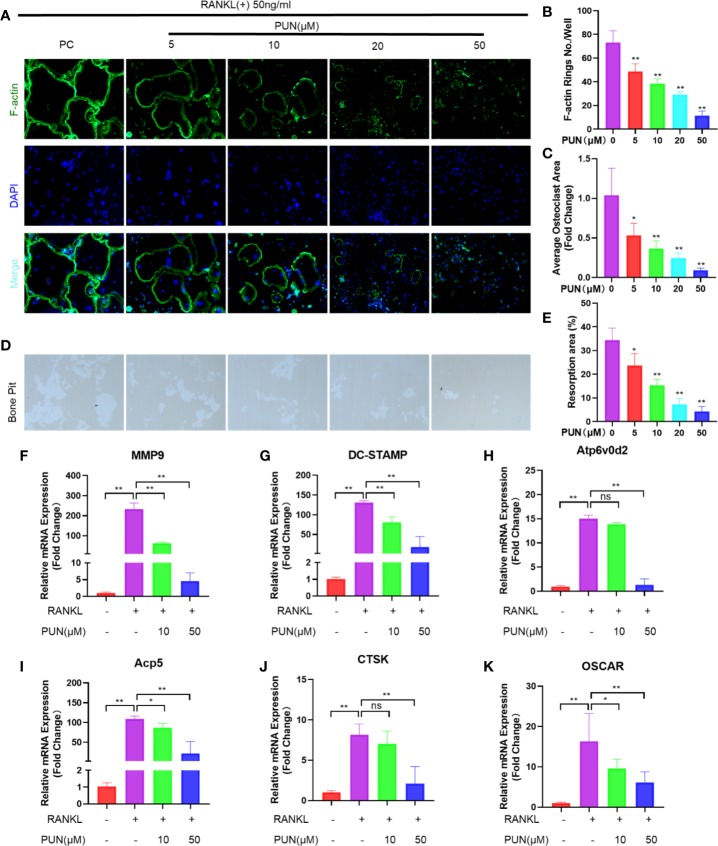
PUN inhibited F-actin rings formation, bone-resorbing activity, and osteoclast-specific genes expression. **(A)** After treatment with or without PUN, RAW 264.7 macrophages were incubated with induction medium for 4 days and then stained for F-actin. F-actin (green) and nuclei (blue) staining of osteoclasts were photographed at the magnification of 320x by fluorescence microscope. **(B)** Quantification of the F-actin ring number per well. **(C)** Quantification of the relative area of osteoclast. **(D)** RAW 264.7 macrophages were plated on bone resorption plates and cultured with induction medium containing 50 ng/ml RANKL together with various concentrations of PUN (0, 5, 10, 20, and 50 μM) for 4 days. Resorption pits were visualized at the magnification of 320× under an inverted microscope. **(E)** Quantification of pits formation area. All bar graphs are presented as mean ± SD. *p < 0.05, **p < 0.01 compared with control group, n = 3 per group. **(F–K)** RAW 264.7 macrophages were incubated in six-well plate in triplicate with induction medium containing 50 ng/ml RANKL and various concentration of PUN for 24 h. The gene copies of MMP9, DC-STAMP, Atp6v0d2, Acp5, CTSK, and OSCAR were quantified by RT-PCR. *p < 0.05, **p < 0.01 compared with RANKL group, n = 6 per group. NS, not statistically significant.

F-actin and DAPI staining were performed to examine osteoclast size and nuclearity. As expected, a well-defined actin belt and representative nuclei (n ≥ 3) were induced by RANKL stimulation (**Figure 2A**). In contrast, PUN treatment significantly decreased the formation and size of F-actin rings in a dose-dependent manner (**Figures 2A–C**).

As mature osteoclasts are capable of resorbing bone, an osteoclastic pit formation assay was used to identify whether PUN inhibited bone resorption. The cells not treated with PUN had greatly increased resorption areas, but PUN treatment dramatically suppressed the resorbing activity, which is in line with the osteoclastogenesis trend (**Figures 2D, E**).

The mRNA levels of several osteoclast-related genes, including matrix metallopeptidase 9 (MMP9), dendritic cell-specific transmembrane protein (DC-STAMP), Atp6v0d2 (ATPase H+ Transporting V0 Subunit D2), Acp5 (acid phosphatase 5, tartrate-resistant), CTSK, and osteoclast-associated receptor (OSCAR), were examined using q-PCR. We observed that these genes were upregulated in RAW 264.7 macrophages when osteoclast differentiation was induced, but these changes were suppressed dose-dependently upon PUN treatment (**Figures 2F–K**).

### PUN Interfered With RANKL-Activated NF-κB and MAPKs Pathways

**Figure 3:**
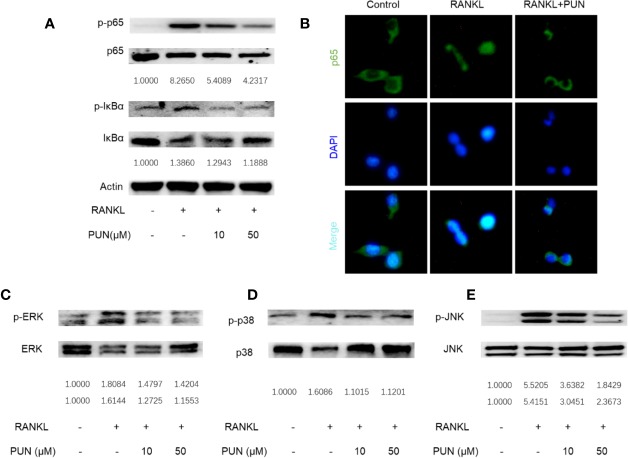
PUN interfered with RANKL-induced activation of NF-κB and MAPKs pathways. After presentative treatment with or without various concentration of PUN (0, 10, and 50 μM) for 6 h, RAW264.7 cells were stimulated by 50 ng/ml RANKL. Then, the cells were collected and lysed for Western blot analysis **(A, C–E)**. The relative grey level of phosphorylated protein of p65, IκBα, p-ERK/MAPK, p-38/MAPK, and JNK to total protein were quantified by using Image Lab software and compared with control group. **(B)** After treated with or without PUN (50 μM), RAW264.7 cells were stimulated by 50 ng/ml RANKL for 1 h and then stained for p65 antibody and secondary antibody with FITC. And the p65 nuclear localization was visualized using an immunofluorescence microscope. n = 3 per group.

NF-κB and MAPK play a crucial role during osteoclastogenesis (Wang et al., 2018; Gao et al., 2019). Western blot and immunofluorescence staining analyses were performed to further determine the effect of PUN on RANKL-induced NF-κB and MAPK pathway activation. The expression levels of phosphorylated inhibitor of NF-κB kinase subunit alpha (IκB-α) and p65 were analyzed to determine NF-κB signaling. In an inactive state, IκB-α combines with NF-κB transcription factors. However, after RANKL stimulation, IκB-α is degraded, and the transcription factors are then activated and released (Gao et al., 2019). As shown in **Figure 3A**, PUN treatment suppressed IκB degradation and p65 activation.

Consistent with the Western blot analysis, the immunofluorescence staining results showed that PUN treatment dramatically interfered with the nuclear accumulation of p65 (**Figure 3B**). For MAPK signaling, three MAPKs, extracellular signal-regulated kinase (ERK), p38, and c-Jun N-terminal kinase (JNK), were phosphorylated and activated after RANKL stimulation (**Figures 3C–E**). We found that PUN treatment interfered with the phosphorylation of ERK (**Figure 3C**), p38 (**Figure 3D**), and JNK (**Figure 3E**). In particular, JNK phosphorylation was significantly inhibited by PUN in a dose-dependent manner (**Figure 3E**). These results suggested that PUN inhibited RANKL-induced osteoclastogenesis by suppressing NF-κB and MAPK pathway activation.

### PUN Prevented Ovariectomy-Induced Bone Loss In Vivo

**Figure 4:**
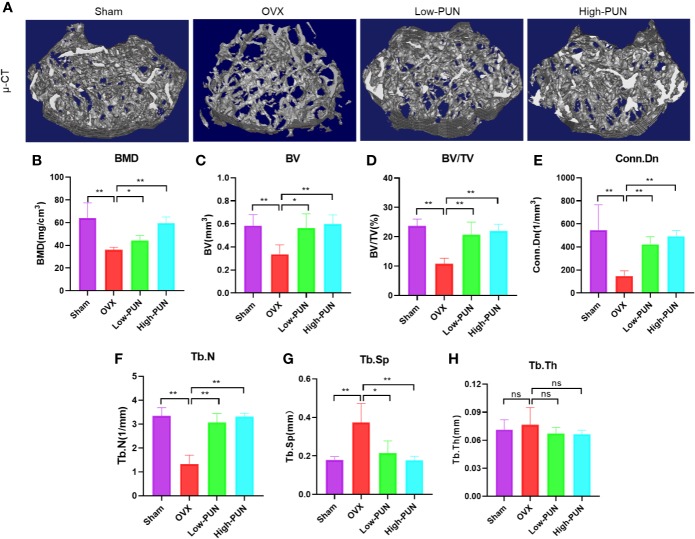
PUN inhibited ovariectomized induced bone loss. **(A)** Micro-CT reconstruction images, **(B)** Bone mineral density (BMD, mg/cm^3^), **(C)** Bone volume (BV, mm^3^), **(D)** Bone volume/tissue volume (BV/TV, %), **(E)** Connectivity density (Conn.Dn, 1/mm^3^), **(F)** Trabecular number (Tb.N, 1/mm), **(G)** Trabecular separation (Tb.Sp, mm),**(H)** Trabecular thickness (Tb.Th, mm). *p < 0.05, **p < 0.01 compared with the OVX group, n = 5 per group. NS, not statistically significant.

Inspired by the encouraging in vitro effects of PUN on osteoclastogenesis, we next investigated the osteoprotective activity of PUN in an experimental ovariectomy (OVX)-induced osteoporosis mouse model. **Figure 4A** illustrates that OVX induced osteoporosis bone loss in murine femurs, but this change was dramatically prevented by PUN treatment. Quantitative analysis of bone parameters showed that the BMD was significantly decreased in the OVX group compared with the sham group (36.085 ± 2.323 mg/cm^3^ vs 64.021 ± 13.456 mg/cm^3^, respectively), but PUN treatment prevented the decrease in BMD (44.169 ± 4.307 mg/cm^3^), especially for the high PUN group (59.393 ± 5.582 mg/cm^3^) (**Figure 4B**). In addition, other parameters, such as the BV, BV/TV, Conn.Dn, Tb.N, and Tb.Sp showed that PUN eased OVX-induced osteoporosis (**Figures 4C–G**). However, in the current study, there was no change in Tb.Th (**Figure 4H**).

**Figure 5:**
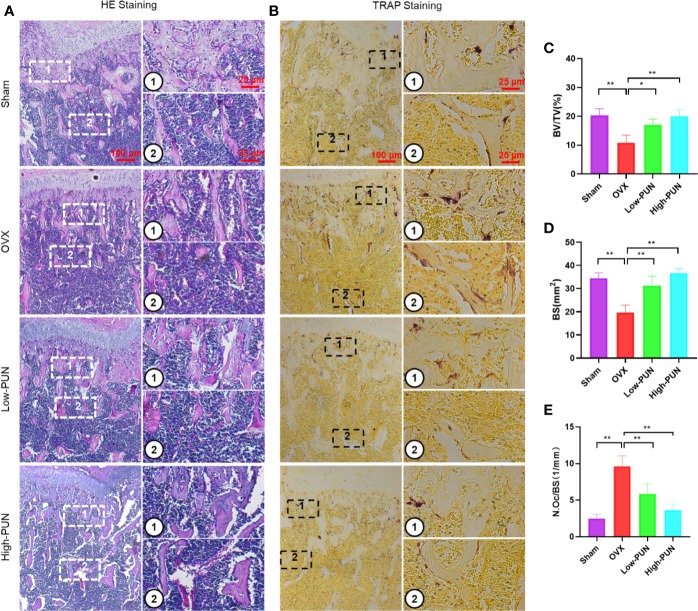
PUN inhibited ovariectomized induced bone loss and reduced osteoclast number. **(A)** H&E staining, **(B)** TRAP staining, **(C)** Quantitative analyses of histomorphometric bone parameters of BV/TV (%), **(D)** Quantitative analyses of histomorphometric bone parameters of BS (mm^2^), **(E)** Quantitative analyses of number of TRAP-positive osteoclasts/bone surface (N.Oc/BS, 1/mm). *p < 0.05, **p < 0.01 compared with the OVX group, n = 5 per group. Scale bar, 100 μm, 25 μm.

Consistent with the micro-CT results, a histological examination showed that the bone loss induced by OVX was reduced by PUN. H&E and TRAP staining were used to determine the osteoprotective activity of PUN treatment (**Figures 5A, B**). PUN-treated mice greatly maintained BV and bone surface compared with those in the OVX group, which was demonstrated by H&E staining quantification (**Figures 5C, D**). Meanwhile, PUN treatment dramatically decreased the average number of osteoclasts/bone surface (N.Oc/BS,1/mm), compared with no PUN treatment in the OVX group (**Figure 5E**).

### PUN Inhibited the Macrophage Proinflammatory Phenotype and Proinflammatory Cytokine Gene Expression

**Figure 6:**
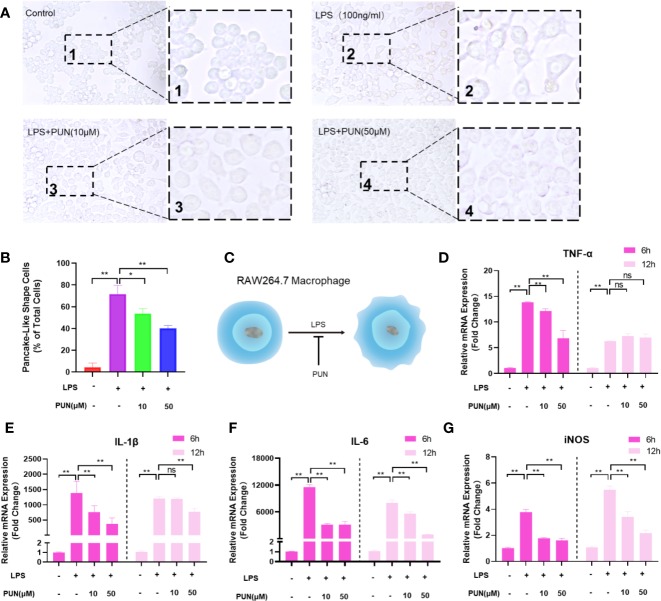
PUN suppressed LPS induced inflammation. **(A)** After presentative treatment with or without various concentration of PUN (0, 10, 50 μM) for 6 h, RAW264.7 cells were stimulated by 100 ng/ml LPS for 24 h. Pancake-like shape cells were visualized at the magnification of 320× under an inverted microscope. **(B)** Quantification of the percentage of pancake-like shape cells. **(C)** The schematic diagram of the effect of PUN on the cellular shape of RAW 264.7 macrophages. **(D–G)** After treated with or without PUN, RAW 264.7 macrophages were incubated in six-well plate with 100 ng/ml LPS and various concentration of PUN for 6 or 12 h. The pro-inflammatory cytokines gene copies of TNF-α, IL-1β, IL-6, and iNOS were quantified by RT-PCR. *p < 0.05, **p < 0.01 compared with LPS group, n = 6 per group. NS, not statistically significant.

Both histopathological analysis and micro-CT imaging demonstrated that PUN relieved OVX-induced bone loss. We confirmed that PUN directly inhibited osteoclastogenesis *in vitro*. Growing evidence indicates that inflammation plays an important role during osteoclast formation and bone resorption (Zhao et al., 2016). Therefore, we next examined whether PUN has a direct inhibitory effect on inflammation. Previous studies have demonstrated that macrophages undergo morphological alterations after stimulation with different molecular signals (McWhorter et al., 2013; Wu et al., 2019). **Figure 6A** illustrates that macrophages appeared round and had a clustered morphology in the absence of LPS and PUN. When stimulated with LPS, the macrophages displayed a round plate shape and loosely compact cells (McWhorter et al., 2013). With the addition of PUN, fewer macrophages with plate shapes and more clustered cells could be observed (**Figures 6A, B**). The schematic diagram (**Figure 6C**) illustrated the effect of PUN on the cellular shape of macrophages.

To further identify the inhibitory effect of PUN on inflammation, RAW 264.7 macrophages were seeded in six-well plates and treated with 100 ng/ml LPS for 6 h or 12 h in the presence or absence of various concentrations of PUN as indicated. Then, quantitative PCR was used to examine proinflammatory cytokine mRNA levels. Several proinflammatory genes, including IL-1β, IL-6, TNF-α, and iNOS, were upregulated during LPS induction, but their expression was inhibited following the addition of PUN (**Figures 6D–G**), except for the TNF-α changes in the 12 h group. These findings indicated that PUN inhibited proinflammatory reactions.

### PUN Interfered With LPS-Induced NF-κB and MAPK Pathway Activation

**Figure 7:**
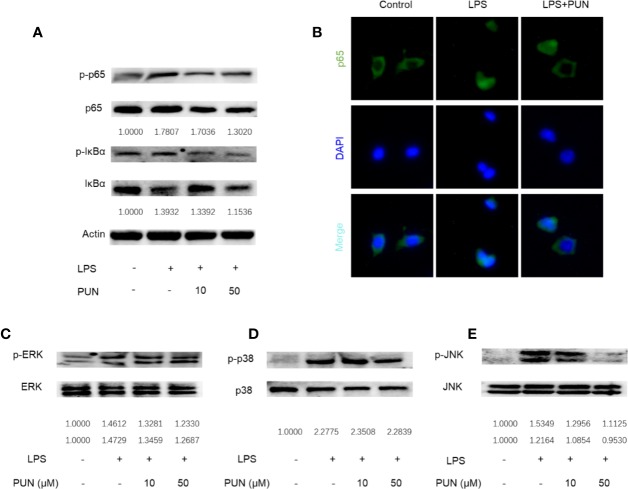
PUN interfered with LPS-induced activation of NF-κB and MAPK pathways. After presentative treatment with or without various concentration of PUN (0, 10, 50 μM) for 6 h, RAW264.7 cells were stimulated by100 ng/ml LPS. Then, the cells were collected and lysed for Western blot analysis **(A**, **C–E)**. The relative grey level of phosphorylated protein of p65, IκBα, p-ERK/MAPK, p-38/MAPK, and JNK to total protein were quantified by using Image Lab software and compared with control group. **(B)** After treated with or without PUN (50 μM), RAW264.7 cells were stimulated by 100 ng/ml LPS for 1 h and then stained for p65 antibody and secondary antibody with FITC. The p65 nuclear localization was visualized using an immunofluorescence microscope.

In immunity and inflammation, the NF-κB and MAPK pathways play a major role in regulating the main proinflammatory mediator genes (Oeckinghaus et al., 2011; Kyriakis and Avruch, 2012). We then investigated the LPS-induced activation of the NF-κB and MAPK signaling pathways. The Western blot results showed that LPS stimulation induced the phosphorylation and activation of NF-κB, while PUN treatment reduced the expression levels of p-IκBα and p-p65 (**Figure 7A**). Consistent with Western blot analyses of p65, nuclear accumulation of p65 was observed using immunofluorescence staining when RAW 264.7 cells were stimulated with LPS, whereas PUN treatment inhibited p65 activation (**Figure 7B**). We also found that three MAPKs, ERK, p38, and JNK, were phosphorylated upon LPS stimulation, but PUN treatment dramatically decreased the levels of p-JNK (**Figures 7C–E**). These results showed that PUN inhibited LPS-induced NF-κB and MAPK pathway activation.

## Discussion

Postmenopausal osteoporosis is further increasing as the population ages (Ihn et al., 2019). Although there are plenty of effective anti-osteoporotic drugs, they unfortunately have secondary effects, including increased risk of breast cancer, myocardial ischemia, thromboembolic disease, and atypical femur facture (Tella and Gallagher, 2014; Black and Rosen, 2016; Gennari et al., 2016). Therefore, it is urgent to discover more effective and safer drugs for osteoporosis therapy. In the present study, we found that PUN, which is found mainly in pomegranates, plays profound anti-osteoporotic roles by inhibiting both osteoclastogenesis and inflammation in OVX mice.

PUN, an ellagitannin that is the most abundant polyphenol, shows significant pharmacological activity, including antioxidant, anticancer, and anti-inflammatory activities (Johanningsmeier and Harris, 2011; Khwairakpam et al., 2018). A previous study reported that PUN can attenuate osteoclast differentiation by blocking the AKT and JNK pathways (Iwatake et al., 2015). In the current study, we observed that PUN reversed RANKL-induced osteoclastogenesis and LPS-induced inflammation. Additionally, we showed that PUN mitigated bone loss in an OVX-induced osteoporosis model by suppressing osteoclast differentiation.

Osteoclasts are unique cells that play a critical role in maintaining bone homeostasis and normal bone density. It has also been demonstrated that osteoclasts are crucial in osteoporosis (Jacome-Galarza et al., 2019; Lee et al., 2019). Excessive osteoclastogenesis and bone resorption result in changes in trabecular bone mass and microstructure, leading to the potential risk of fracture (Tella and Gallagher, 2014; Lin et al., 2016). Our *in vitro* results showed that PUN treatment inhibited osteoclastogenesis in the early stage and interfered with F-actin ring formation and the bone resorptive activity of osteoclasts dose-dependently. Our results also showed that osteoclast-related genes (Acp5 and OSCAR and four functional factors (Atp6v0d2, DC-STAMP, CTSK, and MMP9) were also downregulated during osteoclast differentiation. Iwatake’s study reported that PUN can attenuate osteoclast differentiation *in vitro* (Iwatake et al., 2015), which is in line with our results in the current study. Moreover, in our *in vivo* study, we observed that PUN potently protected against bone destruction, especially in the high PUN group, according to micro-CT and histopathological analysis. Additionally, TRAP staining showed that PUN treatment decreased the osteoclast number and prevented bone loss by inhibiting osteoclast differentiation. Collectively, these results demonstrated that PUN treatment alleviated OVX-induced bone loss by inhibiting osteoclastogenesis.

On the basis of the etiology and pathogenesis of osteoporosis, the current osteoporosis therapies are focused mainly on antiresorptive drugs and anabolic agents (Black and Rosen, 2016; Gennari et al., 2016). However, long-term side effects are found when they are used. Recent findings led to a research focus on different proinflammatory factors, as well as their role in osteoclastogenesis (Pietschmann et al., 2016; Michalski and McCauley, 2017). Estrogen withdrawal will lead to an increase in inflammatory cytokines, and these mediators majorly contribute to excessive osteoclastogenesis and bone resorption in menopause-associated bone loss (Zhao et al., 2016; Pietschmann et al., 2016; Michalski and McCauley, 2017). As PUN has considerable anti-inflammatory effects (Wang et al., 2019), we wondered whether PUN can suppress inflammation in osteoporosis. We found that PUN decreased the numbers and reversed the morphological alterations of proinflammatory macrophages, as well as suppressed the production of inflammatory mediators in LPS-stimulated RAW 264.7 macrophages dose-dependently *in vitro*. This study showed the benefit of PUN in osteoporotic bone loss by inhibiting inflammation, as well as osteoclastogenesis.

**Figure 8:**
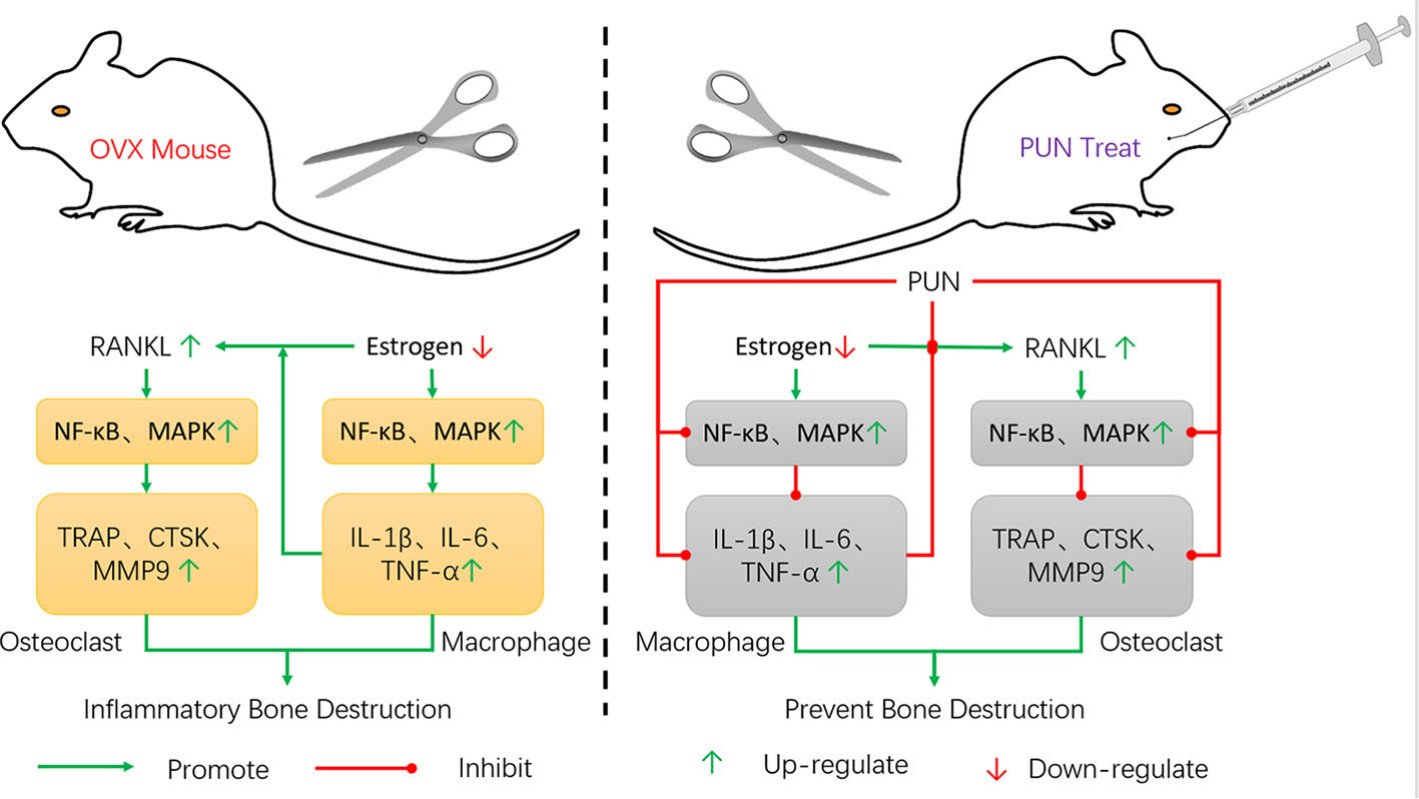
Schematic model of the signal mechanisms underlying the inhibitory effect of PUN on OVX-induced osteoporosis.

The NF-κB and MAPK pathways, whose activation is induced by RANKL, are major signaling pathways regulating osteoclastogenesis and maturation (Wang et al., 2018; Chen et al., 2019). Our findings illustrated that PUN treatment interfered with the NF-κB and MAPK pathways, including IκBα degradation and p65, p38, ERK, and JNK phosphorylation. In immunity and inflammation, the NF-κB and MAPK pathways also play an important role in regulating the gene levels of the primary proinflammatory mediators (Oeckinghaus et al., 2011; Kyriakis and Avruch, 2012; Gao et al., 2019). Our findings showed that NF-κB activation by LPS stimulation was significantly altered by PUN. PUN also dramatically interfered with the phosphorylation of JNK. These findings indicated that PUN achieved anti-osteoclastic and anti-inflammatory effects by suppressing NF-κB and MAPK signaling activation (**Figure 8**). However, we cannot exclude the possibility that PUN might affect osteoblastic bone formation. Study has reported that pomegranate peel extract prevents bone loss by stimulating osteoblastic differentiation in the model of osteoporosis (Spilmont et al., 2015), but the contribution of PUN and the molecular mechanisms remain unclear, thus, further investigations are needed to examine the effects of PUN on osteoblastic function. And the effects of PUN on are very similar to estrogen therapy, it would be of interest to determine whether PUN for some reason is a phytoestrogen, which still remains to be further investigated.

The present study demonstrated that PUN, which is found mainly in the pomegranate fruit, played profound antiosteoporosis roles by attenuating the formation and differentiation of osteoclasts and inhibiting proinflammatory cytokines. Mechanistically, PUN maintained bone mass *via* suppressing the activation of the NF-κB and MAPK signaling pathways. PUN may be a candidate drug for osteoporosis and other chronic osteoclastogenesis/inflammation-related disorders.

## Data Availability Statement

The raw data supporting the conclusions of this article will be made available by the authors, without undue reservation, to any qualified researcher.

## Ethics Statement

All animal experimental procedures were performed with the approval of the Ethics Committee of the First Affiliated Hospital of Soochow University.

## Author Contributions

WW, JB, WZ, DG, and JZ contributed to the conception of the work. WW, JB, WZ, GG, QW, and XL contributed to the experiments. WW, JB, WZ, NL, YG, and ML contributed to the data acquisition. WW and DG wrote the manuscript. WX, HY, YX, and JZ revised the manuscript. All authors contributed to the final approval of the manuscript.

## Funding

This research was supported by the National Nature Science Foundation of China (81873990, 81873991, 81672238, 81472077, and 81372018), the Jiangsu Provincial Medical Youth Talent (QNRC2016751), the Natural Science Foundation of Jiangsu province (BK20180001), the Priority Academic Program Development of Jiangsu Higher Education Institutions (PAPD).

## Conflict of Interest

The authors declare that the research was conducted in the absence of any commercial or financial relationships that could be construed as a potential conflict of interest.
